# Clinical practice guidelines within the Southern African development community: a descriptive study of the quality of guideline development and concordance with best evidence for five priority diseases

**DOI:** 10.1186/1478-4505-10-1

**Published:** 2012-01-05

**Authors:** Tamara Kredo, Annette Gerritsen, Johan van Heerden, Shaun Conway, Nandi Siegfried

**Affiliations:** 1 South African Cochrane Centre, South African Medical Research Council, Cape Town, Western Cape, South Africa; 2 Epi Result Consultancy, Louis Trichardt, Limpopo Province, South Africa; 3 Benguela Health, Centurion, Gauteng, South Africa; 4 Southern African Regional Programme for Access to Medicines, Johannesburg, Gauteng, South Africa

**Keywords:** clinical practice guidelines, quality, evidence-based, alignment

## Abstract

**Background:**

Reducing the burden of disease relies on availability of evidence-based clinical practice guidelines (CPGs). There is limited data on availability, quality and content of guidelines within the Southern African Development Community (SADC). This evaluation aims to address this gap in knowledge and provide recommendations for regional guideline development.

**Methods:**

We prioritised five diseases: HIV in adults, malaria in children and adults, pre-eclampsia, diarrhoea in children and hypertension in primary care. A comprehensive electronic search to locate guidelines was conducted between June and October 2010 and augmented with email contact with SADC Ministries of Health. Independent reviewers used the AGREE II tool to score six quality domains reporting the guideline development process. Alignment of the evidence-base of the guidelines was evaluated by comparing their content with key recommendations from accepted reference guidelines, identified with a content expert, and percentage scores were calculated.

**Findings:**

We identified 30 guidelines from 13 countries, publication dates ranging from 2003-2010. Overall the '*scope and purpose' *and '*clarity and presentation' *domains of the AGREE II instrument scored highest, median 58%(range 19-92) and 83%(range 17-100) respectively. '*Stakeholder involvement' *followed with median 39%(range 6-75). '*Applicability'*, '*rigour of development' *and '*editorial independence' *scored poorly, all below 25%. Alignment with evidence was variable across member states, the lowest scores occurring in older guidelines or where the guideline being evaluated was part of broader primary healthcare CPG rather than a disease-specific guideline.

**Conclusion:**

This review identified quality gaps and variable alignment with best evidence in available guidelines within SADC for five priority diseases. Future guideline development processes within SADC should better adhere to global reporting norms requiring broader consultation of stakeholders and transparency of process. A regional guideline support committee could harness local capacity to support context appropriate guideline development.

## Introduction

Clinical practice guidelines bridge the gap between policy and practice and should be based on up-to-date, high quality research findings [1,2]. Reducing the burden of disease in resource-poor settings relies on the availability of such evidence-based clinical practice guidelines [3]. Gaps in these guidelines may impact on the health of the public they are meant to serve. These omissions may be a result of opinion-based rather than evidence-based guidance; recommendations from guideline development groups with undisclosed conflicts of interest; or poor planning for implementation of a guideline [4-8]. There is little data regarding the quality and content of guidelines in Southern Africa, a region facing serious health issues including poorly contained communicable diseases, increasing non-communicable diseases and under-resourced, often poorly managed health systems. This demands increasing attention from both development agencies and researchers to support research aimed at strengthening guidelines and policy [9].

Several guideline appraisal tools have been developed to assess the quality of guidelines [10]. Of these, the Appraisal of Guidelines for Research and Evaluation (AGREE) tool has been validated and is most widely accepted [11-13]. None of the available instruments assesses the clinical content of the guideline or the quality of the supporting evidence [10]. It is important to develop methodology for assessing alignment of published guidelines with current best evidence.

Our project aimed to evaluate clinical practice guidelines (CPGs) from the Southern African Development Community (SADC) member states, for priority diseases, with respect to availability, quality and alignment with current reference guidelines. This study formed part of a larger programme, the Southern African Regional Programme on Access to Medicines (SARPAM) for harmonising CPGs and essential medicine lists for reforming regional procurement.

## Methods

### Prioritising guidelines

We considered two issues: firstly, priority diseases should be representative of the following key components: adult & paediatric conditions; communicable & non-communicable diseases; chronic and acute onset diseases; maternal health; hospital-based & primary health care conditions. Secondly, time and feasibility limited our selection to five diseases. The following conditions were prioritised by the SARPAM study team: HIV in adults; malaria in children and adults; essential hypertension in primary care; pre-eclampsia; and diarrhoea in children.

### Search strategy for guidelines

A public health clinician used an electronic search and e-mail enquiry strategy to obtain the CPGs for each SADC country: Angola, Botswana, the Democratic Republic of Congo (DRC), Lesotho, Malawi, Mauritius, Mozambique, Namibia, Seychelles, South Africa, Swaziland, United Republic of Tanzania, Zambia and Zimbabwe. The search strategy incorporated possible document and disease terms which were added to the country names (Table 1). Medline and Google were searched using an iterative approach. We contacted Ministries of Health via the SADC secretariat. Only English language guidelines were accepted for this evaluation.

**Table 1:** Search terms used for finding clinical practice guidelines within SADC

Search concepts	Search terms
**Medical terms**	HIV; AIDS; ART; ARV; HAART; Anti-retroviral treatment/therapy; Communicable disease/s Malaria Diarrhoea; acute; paediatric; child/ren Hypertension: cardiovascular disease; CVD Pre-eclampsia: hypertension; pregnancy

**Countries**	Angola, Botswana, The Democratic Republic of Congo, Lesotho, Malawi, Mauritius, Mozambique, Namibia, Seychelles, South Africa, Swaziland, United Republic of Tanzania, Zambia and Zimbabwe

**Clinical practice guidelines**	Standard treatment guideline/s; STG/s; Standard Treatment; Treatment; Treatment guideline/s

**Essential medicine lists**	Essential medicine list; EML; Essential drug list; EDL; Central medical store; procurement list; CMS; Medicine procurement list

**Ministries of health**	Department of Health; DOH; Ministry of Health; MOH; National Aids Commission; Aids Commission; NAC

### AGREE II instrument

Two reviewers (TK, AG) independently evaluated the global quality of the five CPGs for each of the SADC states and the reference guidelines using AGREE II. This tool is a recently revised and validated version of the AGREE instrument [14-18]. AGREE II contains 23 key quality items categorised in six domains scored with a 7-point Likert scale. Standardised guideline domain scores were calculated by summing scores of individual items and standardising the total as a percentage of the maximum possible score for that domain. The six domain scores are independent and were not aggregated into a single quality score. Uncertainties in the application of AGREE II were resolved in consultation with a third investigator (NS). We used Microsoft™ Excel to record the scores. As the data was nonparametric, we calculated a median (range) for each domain across countries to provide overall results.

### Alignment with reference standard guidelines and expert opinion

We invited one South Africa-based content expert for each of the five priority areas to give input on this project. The current gold standard reference guideline for each topic was identified in consultation with the content expert [19-26]. The key items of evidence that should appear in a current guideline on that topic were then extracted (TK) and the list judged and summarised by the relevant content expert. These lists represent the recommendations against which alignment with current best evidence could be checked with the in-country guideline. The list of recommendations from each of the reference guidelines was assigned a point score according to the number of recommendations that should be present to indicate alignment with the reference guideline. For example, 25 key items were identified for HIV guidelines from the WHO 2010 guidelines for the management of HIV/AIDS in adults and adolescents(19); all current HIV management guidelines should include these and would score 100% if all 25 points were identified (Table 2; tables for list of recommendations for all reference guidelines are available on request). All in-country CPGs were assessed for concordance with the recommendations list. There were no specific weightings for the individual recommendations, as each item is considered a key item for inclusion in current guidelines on those topics. For each of the five diseases, we summarised the concordance of the CPGs using a percentage score and noted any differences.

**Table 2:** Key recommendations from the reference guideline for the management of HIV in adults

	WHO 2010	Details	Points
**Recommendation 1**	When to start	- CD4 count < 350 - WHO Clinical stage 3 and 4 irrespective of CD4 count	2

**Recommendation 2**	What to start	- AZT+3TC +EFV - AZT+3TC+NVP - TDF+3TC/FTC+EFV - TDF+3TC/FTC+NVP	4

**Recommendation 3**	ART for HIV/TB	- Start ART in all patients with TB - Start TB treatment first - Prefer EFV - Start ART within 2-8 weeks of starting TB treatment - If CD4 count < 200, start ART within 2 weeks	5

**Recommendation 4**	ART for HIV/Hep B	- Start ART in all patients who require treatment for their Hepatitis B - Start TDF and 3TC/FTC	2

**Recommendation 5**	ART for pregnancy	- Start ART in all pregnant women if CD4 count < 350 - Start ART in all women with clinical stage 3 or 4 disease irrespective of CD4 count - AZT preferred in pregnancy - EFV or NVP can be used - Do not start EFV in first trimester	6

**Recommendation 6**	When to switch - (note: if VL 5000 or less, will be accepted e.g. 1000)	- VL > 5000copies/mL on at least two occasions - Use CD4 count if VL not available	2

**Recommendation 7**	Second line ART (note: if any one of the protease inhibitors included, will accept)	- Boosted PI + 2 NRTIs recommended - Atazanavir/ritonavir or Lopinavir/ritonavir or darunavir/ritonavir recommended - If TDF used in first line, use AZT/D4T next - AND if AZT/D4T used in first line, use TDF in second line	4

ART = antiretroviral therapy; TB = tuberculosis; Hep B = hepatitis B; VL = viral load; PI = protease inhibitor; NRTIs = nucleoside reverse transcriptase inhibitors; TDF = tenofovir; AZT = zidovudine; D4T = stavudine; EFV = efavirenz; NVP = nevirapine

## Results

### Search results

The search was conducted between June and October 2010, including feedback from a SADC secretariat meeting in September 2010. The MEDLINE search yielded no results. Using Google™ and personal contacts the search yielded 30 guidelines from 13 SADC states (Table 3). The publication dates of the available guidelines ranged from 2003 to 2010. HIV guidelines were available from 13 of the 14 states; three were in languages other than English. Malaria treatment guidelines were available from 13 of the states, two were not in English, leaving 11 evaluable for this review, three of which were sections within other CPGs. Hypertension guidelines were available from nine countries, only two of which were disease-specific guidelines (South Africa and Mauritius)[27]. We did not locate guidelines dedicated to management of diarrhoea in children or pre-eclampsia. We did evaluate the broader primary care CPGs for these diseases in seven member states for the former condition and eight member states for the latter.

**Table 3:** SADC member state guidelines and the reference standard guidelines

SADC countries	HIV therapy in adults	Malaria therapy in adults and children	Diarrhoea therapy in children	Hypertension therapy in primary care	Pre-eclampsia therapy	Primary care clinical practice guideline^iv^
**Angola**	N/A^i^	N/A	N/A	N/A	N/A	N/A

**Botswana**	2008^ii^	2007	N/A	N/A	N/A	N/A

**Democratic Republic of Congo**	2005 (French)^iii^	2005 (French)	N/A	N/A	N/A	N/A

**Lesotho**	2010 draft	N/A	N/A	N/A	N/A	2005

**Malawi**	2008	N/A	N/A	N/A	N/A	2009

**Mauritius**	2009 (French)	?date	N/A	?date	N/A	N/A

**Mozambique**	2009 (Portuguese)	2006 (Portuguese)	N/A	N/A	N/A	N/A

**Namibia**	2009	2005	N/A	N/A	N/A	2010

**Seychelles**	N/A	N/A	N/A	N/A	N/A	2003

**South Africa**	2010	2009	N/A	2006	N/A	2008

**Swaziland**	2006	2009	N/A	N/A	N/A	N/A

**Tanzania**	2005	2006	N/A	N/A	N/A	2007

**Zambia**	2007	N/A	N/A	N/A	N/A	2008

**Zimbabwe**	2010 draft	N/A	N/A	N/A	N/A	2006

**Standard reference guideline**	World Health Organization, 2010^19^	World Health Organization, 2010^20^	World Health Organization, 2005^22,23^	National Institute for Clinical Excellence 2004^24-26^	Royal College of Obstetricians Gynaecologists, 2006^21^	

^i ^N/A indicates that the guideline was not available during the search period

^ii ^Dates of publication indicated where was available. Where date was not clear we have indicated this with '?date'

^iii ^The language of the guideline-if other than English-is indicated in brackets

^iv ^Primary care guidelines were used to assess alignment when no disease-specific guideline existed

### Summary of AGREE II findings

**Figure 1:**
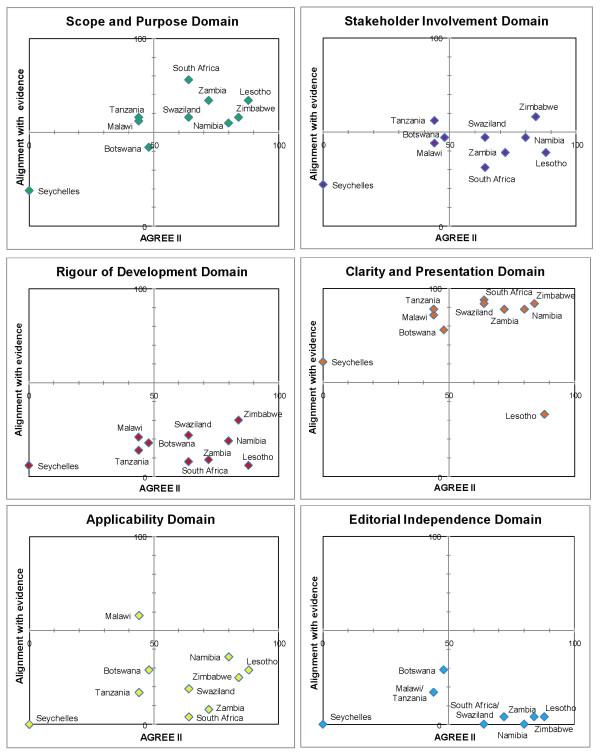
**Matrix of AGREE II and alignment with evidence-HIV guidelines within SADC**.

**Figure 2:**
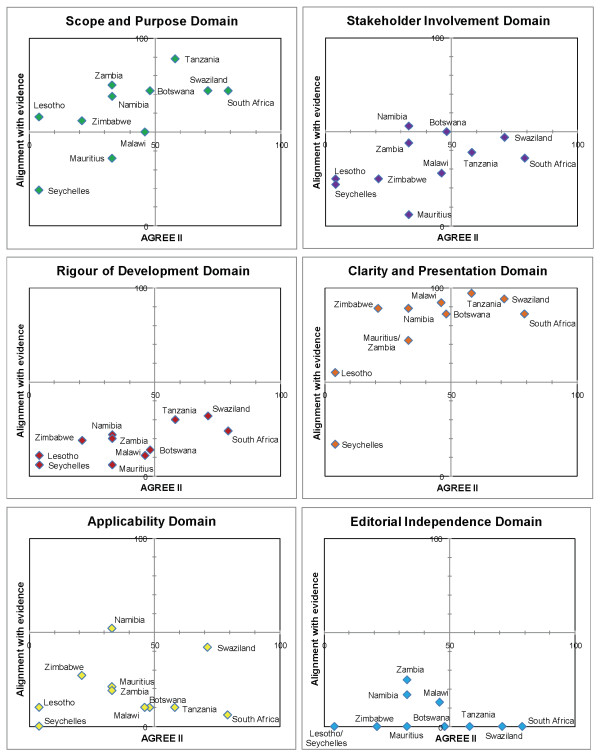
**Matrix of AGREE II and alignment with evidence-Malaria guidelines within SADC**.

**Figure 3:**
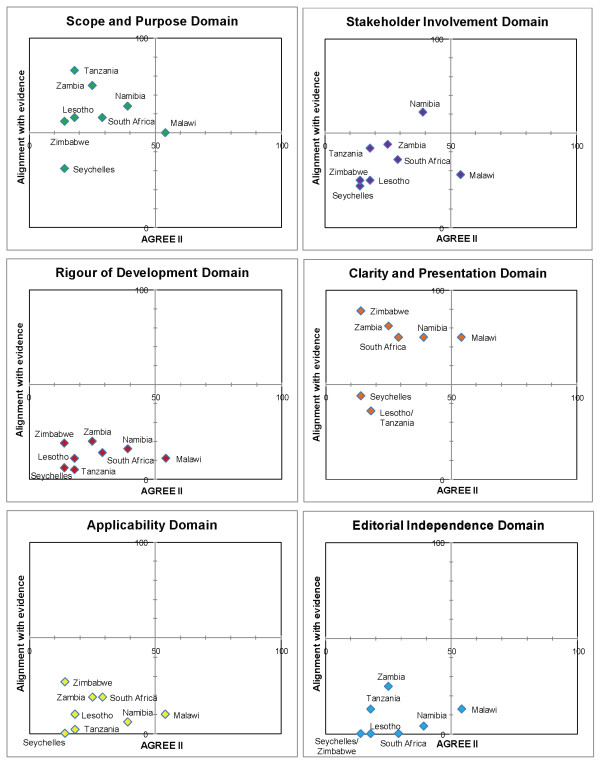
**Matrix of AGREE II and alignment with evidence-Pre-eclampsia guidelines within SADC**.

**Figure 4:**
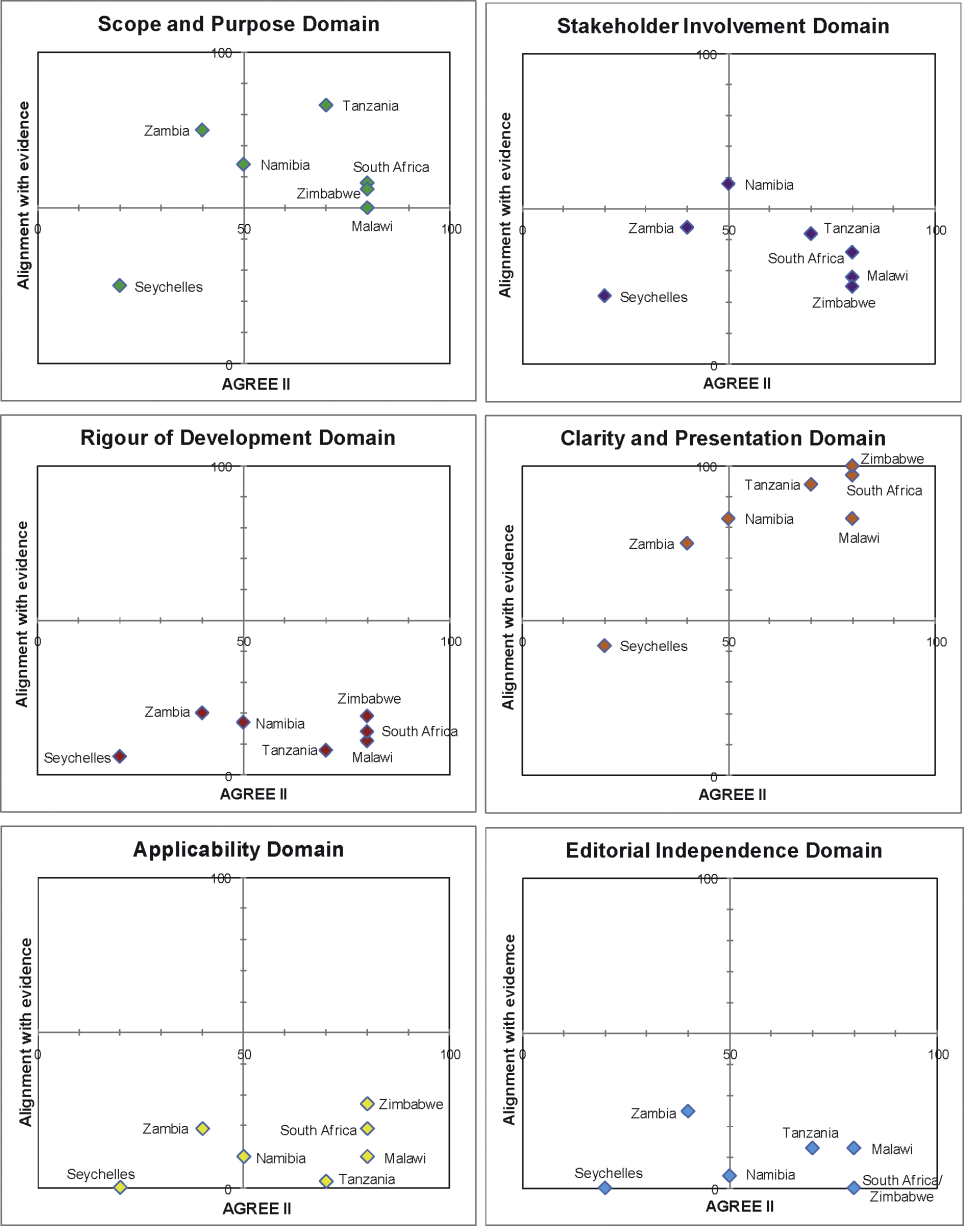
**Matrix of AGREE II and alignment with evidence-Childhood diarrhoea guidelines within SADC**.

**Figure 5:**
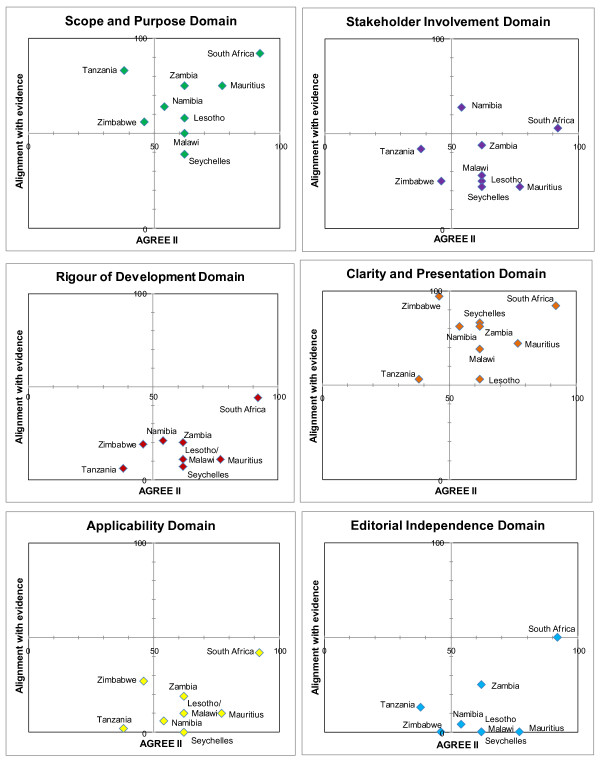
**Matrix of AGREE II and alignment with evidence-Primary care hypertension guidelines within SADC**.

**Figure 6:**
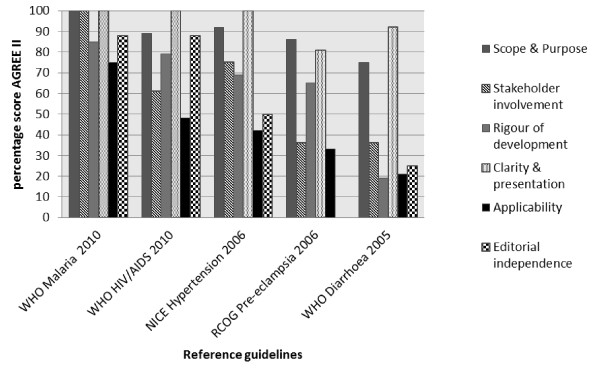
**AGREE II scoring for reference guidelines**.

We present the results according to diseases (Table 4). Matrices are shown which report the intersection of AGREE II by domain and alignment with best evidence (Figures 1, 2, 3, 4 and 5). AGREE II evaluation of the reference guidelines is shown in Figure 6.

**Table 4:** Aggregated AGREE II Scores across diseases [median(range)]

Priority diseases	Domains of AGREE II					
	
	Scope and purpose	Stakeholder involvement	Rigor of development	Clarity and presentation	Applicability	Editorial independence
HIV (n = 10)	57(19-78)	43(22-58)	16(6-30)	88(33-94)	22(0-58)	4(0-29)

Malaria (n = 11)	71(19-89)	38(6-53)	20(6-32)	88(17-97)	15(0-52)	0(0-25)

Pre-eclampsia (n = 8)	58(31-83)	36(22-61)	14(5-20)	75(36-89)	10(0-27)	0(0-25)

Diarrhoea in children (n = 7)	58(25-83)	36(22-58)	14(6-20)	83(42-100)	10(0-27)	4(0-25)

Hypertension (n = 9)	75(39-92)	42(22-64)	11(6-44)	81(53-97)	10(0-42)	4(0-50)

Overall	58(19-92)	39(6-75)	14(5-44)	83(17-100)	10(0-58)	0(0-50)

#### HIV/AIDS

Of the available HIV guidelines, most were disease-specific guidelines, except that from Seychelles, which had an abbreviated guideline on HIV management, forming part of the larger primary care CPG. Two of the guidelines were in the process of being revised after the release of the recent WHO 2010 guideline (Table 3) [19]. The 'clarity and presentation' and 'scope and purpose' domains scored highest with median scores of88% (range 33-94) and 57% (range 19-78) respectively across all countries. 'Rigour of development' and 'editorial independence' were most poorly reported scoring a median score of 16% (range 6-30) and 4% (range 0-29) respectively.

#### Malaria

The 'scope and purpose' and 'clarity and presentation' domains scored highest, median scores of 71% (range 19-89) and 88% (range 17-97), whereas the 'applicability' and 'editorial independence' scored poorly, median 15% (range 0-52) and 0% (range 0-25) respectively.

#### Pre-eclampsia

Eight guidelines were evaluated, including the 2010 draft version of the Namibian CPG. The lowest score was seen for the 'editorial independence' domain, median 0% (range 0-25) and the highest score was seen in the 'clarity and presentation' domain median 75% (range 36-89).

#### Diarrhoea in children

Domains 'scope and purpose', 'stakeholder involvement' and 'clarity and presentation' received highest scores; the lowest score was seen for editorial independence median 4% (range 0-25).

#### Hypertension in adults

The median score for the domain on 'clarity and presentation' was 81% (range 53-97), and the 'scope and purpose' median score was 75% (range 39-92), however the other domains scored largely below 50% with 'editorial independence' scoring lowest, median 4% (range 0-50).

### Alignment of CPGs with reference standards

Key recommendations from the reference guidelines were identified with input from experts in the respective fields.

#### HIV/AIDS

The Zimbabwe (2010) and Lesotho (2010) guidelines, both in draft form, were best aligned with current evidence-based guidelines and expert opinion, achieving > 80% alignment (Figure 1). Older guidelines, such as that from Tanzania (2005), were not well aligned and presented out-dated recommendations such as the use of stavudine as first-line antiretroviral therapy. The guideline from Zambia (2007), achieved good alignment despite having been published prior to the current WHO recommendations. This guideline made provisions for recommending the antiretroviral Tenofovir prior to, but in anticipation of, its availability for first-line therapy in the country.

#### Malaria

The five malaria guidelines that were part of a larger primary care CPG were limited in their scope and generally did not provide comprehensive management advice (Figure 2). Older primary care CPGs were less likely to be in-line with current evidence and tending to score lower in their alignment (Lesotho 2005, Seychelles 2003). Guidelines from South Africa (2009) and Swaziland (2009) were best aligned with current evidence-certain recommendations that differed from reference standard advice were justified due to local cost or regulatory constraints, rather than lack of adherence to best standards (e.g. use of parenteral quinine rather than artesunate in South Africa where the drug is not yet registered by the National Regulatory Authority).

#### Pre-eclampsia

Most guidance regarding the management of pre-eclampsia was brief, scoring poorly overall. Malawi (2009) was most recently published and was current in its recommendations and scored above 50% (Figure 3).

#### Diarrhoea

Diarrhoea management has not changed significantly in the recent years and as such, most CPGs produced after the WHO publication in 2005 (22,23) had fair concordance with recommendations, including the use of zinc in all guidelines. Zimbabwe (2006), South Africa (2008) and Malawi (2009) scored 80% alignment (Figure 4).

#### Hypertension

Two countries with dedicated hypertension guidelines, South Africa (2006) and Mauritius (unknown date), scored best in their alignment with current best evidence [27] (Figure 5). The remaining primary care guidelines did not provide adequate diagnosis and care recommendations hence scoring poorly.

## Discussion

To our knowledge this is the first study to report on the availability and appraisal of quality and content of clinical guidelines for five priority diseases within the SADC region. Of the available guidelines overall scores were poor using the AGREE II assessment-particularly with respect to rigour of development, applicability and editorial independence. Alignment with best evidence was highly variable, with better scores for guidelines that were more recently published and those that were disease-specific rather than sections within larger primary health care CPGs.

### Summary of main findings

HIV and malaria were most likely to have disease-specific guidelines which may reflect the global funding streams and political impetus targeting these conditions. The other priority diseases occupied sections within larger primary care CPGs. Our review found that the sections within other manuals that we evaluated were not comprehensive and provided incomplete guidance and were less likely to be up-to-date. Overall, the 'scope and purpose' and 'clarity and presentation' domains of the AGREE II tool were reported most comprehensively. However, the purpose, health question and target users were not explicitly described. Rather, the information was incorporated within the introduction and foreword sections and required extraction in order to identify the scope and objectives.

Most documents employed clear and consistent methods for identifying key recommendations, such as flow diagrams, tables and highlighted text, making the documents accessible for end-users and resulting in good scores in 'clarity and presentation'. The value of the clarity and presentation has been questioned as it does not strictly reflect the internal and external validity of a guideline document [28]. However, the usability of a guideline may impact on the applicability of the document. Target-users are not necessarily trained to discriminate on the quality of the guideline, but may be encouraged to use it if simple to navigate and apply [29].

The remaining four domains scored poorly across all diseases. The guidelines described the 'stakeholder involvement' of multidisciplinary professional groups; however, little was reported about the contribution of primary-care doctors and target patients. This is increasingly recognised as important for assuring that guidelines represent the needs of both the target users and patients. Involving these groups in the guideline development process, for example by pre-testing the guideline, or evaluating and incorporating preferences and values, may aid in securing the successful implementation of the guideline [30,31].

In our study, as in previous studies, 'rigour of development' scored poorly [12]. A minority of guidelines provided references to the primary data and despite this many guidelines were highly aligned with current evidence (Figures 1, 2,3, 4 and 5). A plausible explanation is that the data required to evaluate this domain may exist in supporting documentation, such as appendices, which our search failed to locate. In addition, many of the SADC guidelines base their recommendations on other reference guidelines, such as WHO publications. Within SADC there may be members that have the capacity to appraise, synthesise and apply current evidence but generally it is accepted that few SADC countries are currently equipped with the necessary technical and financial resources. Despite this, had the guidelines we assessed clearly referenced their source guideline, they should have scored higher in this domain. In future similar evaluations, it may be prudent to augment the 'rigour of development' domain to clearly interrogate the source of the guidelines document, including whether it is based on another reference guideline. Many of the guideline documents we evaluated indicated that there would be a process for updating but none were explicit in their methods or the timing of updates. An important finding from this report is the lag between revisions of some of the guidelines with the result that the recommendations are no longer informed by current evidence potentially posing a risk to public health.

The methods necessary to successfully implement the guidelines, were not clearly delineated, hence the low scoring 'applicability domain'. Facilitators and barriers to applying the guidelines should be described to support implementation. The process of defining facilitators and barriers to application should be integrated early in the guideline development process and include professionals proficient in implementation strategies [29].

The low score in the 'editorial independence' domain reflects the poor reporting of potential conflicts of interest of stakeholders and the potential influence of funders in the guideline development process. Although the absence of these declarations does not necessarily imply that inappropriate influences guided the final recommendations, the presence of such declarations ensures that a guideline can be considered trustworthy [8,13,32].

Higher alignment scores were attained when guidelines were dedicated to a specific illness as seen with the malaria, HIV and hypertension guidelines. Gaps in the key recommendations occurred when the guidelines were out-of-date, occurring more frequently in the primary care CPGs. Pre-eclampsia scored poorly for alignment-indicating that the primary care CPGs we evaluated did not reflect current evidence. This condition requires hospital-based care and we did not identify any secondary or tertiary hospital guidelines during this review. Good alignment was achieved in guidelines despite poor scores in the 'rigour of development' domain-indicating a possible mismatch of the tool with the local practice of basing guidelines on WHO or equivalent high quality guidelines.

### Agreement with previous research

A systematic review evaluating 42 guideline appraisal studies, including 626 guidelines, between 1988 and 2007 using the AGREE tool found similar distributions of low and high scores, supporting the notion that the domains within the guidelines that require improvement are similar despite disease or region [12]. Our scores for rigour of development, editorial independence and applicability were substantially lower than those described-indicating areas that require particular attention for future guideline development within SADC. Our study further highlights the need for support to improve the quality of guidelines by implementation of current normative standards of reporting within guidelines such as those developed by established guideline developers [33-35].

### Strengths and limitations

We were mandated by the SADC Secretariat Pharmaceuticals Programme and therefore received cooperation from the ministries within the member states to assist with locating the relevant guidelines. We have attempted to address the research-knowledge gap by communicating a technical summary of the results to the SADC secretariat with specific recommendations for improving the availability, content and quality of CPGs within SADC. Although the AGREE II tool has been adopted widely as the reference tool to be used to evaluate guideline quality, this is the first time, to our knowledge, that it has been systematically applied across several diseases in a number of resource-constrained countries in Africa. This study can therefore contribute to a validation of the AGREE II tool and support uptake in our setting. The assessment of the alignment of the contents of the CPGs in this review was conducted with both published normative standard guidelines, such as WHO guidelines, and the input from experts in the respective fields.

We did not locate all relevant documents given the absence of a central or country-level repository. The outstanding documents would be required to provide a representative baseline analysis for SADC. The guidelines we evaluated included a combination of disease-specific guidelines and sections within larger primary care manuals. These guidelines may not lend themselves to be pooled in analysis, but do provide a true reflection of current guidance of the management of the included diseases. AGREE collaborators recommend that increasing the number of reviewers increases validity [11,19]. Cost and time constraints dictated the feasible number of content experts and reviewers for this evaluation. There is currently no validated method for assessing alignment with evidence; therefore we used a method that will need future review to assess validity. Lack of timely translation prevented us from reviewing the guidelines from French- and Portuguese-speaking countries. This should be addressed in a future evaluation. The overall appraisal of quality of the guidelines would be enhanced by supplementary consultation and interview-based data collection with ministries, giving particular attention to guideline development processes and strategies and the roles of various members of the ministries of health, scientists and technical experts in formulating the guidelines [10]. Although the AGREE II tool may be applied across regions and settings, our experience suggests that 'rigour of development' domain may have scored more poorly than warranted, as the majority of SADC guidelines rely on guidance from the WHO, and therefore do not reference primary research as the domain requires. For this reason we recommended that this domain be amended for future evaluations for use in our setting.

### What have we learned?

It is important that gaps in the availability of CPGs be identified and addressed. A repository of all guidelines in an accessible database will facilitate access for all SADC member states. It will facilitate cataloguing of guidelines and enable identification of those that may be relevant but missing or out-of-date. This could be done in collaboration with organisations such as the Guideline International Network [36].

There may be value in creating a SADC guideline support committee, through the SADC Pharmaceutical Programme, to assist all member states to adapt, maintain and update in-country guidelines of high standard [37]. This will facilitate that expertise in guideline development be shared. This committee may enlist expertise in reviewing current evidence with regards its applicability and generalisability to local healthcare needs. Such a committee should include, amongst others, the following relevant stakeholders-content experts; funders; policy makers; public health professionals; physicians, nurses and pharmacy staff; patient representative groups and an external review committee. Collaboration with experts in the field of guideline development could support capacity development and aid the process of bridging research and practice.

The value of this review has been to identify specific gaps in the quality and content of the guidelines within SADC. There is increasing awareness that the transfer of research evidence into policy and practice is a complex issue, sensitive to the context of each country. In order to inspire confidence in the quality and evidence-base of guideline recommendations and in the transparency of the development process, each newly developed or updated guideline should adhere to the recommended reporting norms currently in use globally.

## Non-standard abbreviations

CPG: clinical practice guideline; AGREE: Appraisal of Guidelines for Research and Evaluation.

## Competing interests

The authors declare that they have no competing interests.

## Authors' contributions

TK, NS, SC and JvH designed the protocol. AG, TK scored the guidelines using AGREE, NS provided input during process. TK evaluated alignment with key evidence-based recommendations from reference guidelines and content experts. TK drafted the manuscript, NS, JvH, AG and SC contributed to the final report. All authors have read and approved the final manuscript.
